# Economic Burden of Rheumatoid Arthritis in Low‐ and Middle‐Income Countries: Systematic Review and Meta‐Analysis

**DOI:** 10.1002/acr.25627

**Published:** 2025-11-22

**Authors:** Tadesse Gebrye, Chidozie E. Mbada, Clara T. Fatoye, Faatihah Niyi‐Odumosu, Ushotanefe Useh, Zalmai Hakimi, Francis Fatoye

**Affiliations:** ^1^ Manchester Metropolitan University Manchester United Kingdom; ^2^ University of the West of England Bristol United Kingdom; ^3^ North‐West University Potchefstroom South Africa; ^4^ Swedish Orphan Biovitrum AB Stockholm Sweden; ^5^ Manchester Metropolitan University, Manchester, United Kingdom, and North‐West University Potchefstroom South Africa

## Abstract

**Objective:**

The aim of this systematic review was to synthesize the economic impact of rheumatoid arthritis (RA) on households, health systems, and society in low‐ and middle‐income countries (LMICs).

**Methods:**

Electronic databases such as PubMed, Web of Science, and CINAHL were searched using keywords related to RA and cost of illness. Eligible studies were required to report RA‐related costs, be conducted in LMICs, and be published in English. Quality appraisal of the included studies was conducted using the Newcastle–Ottawa Scale for cohort studies. A narrative synthesis and meta‐analysis of findings was conducted.

**Results:**

A total of 5,134 studies was initially identified for screening. After removing 1,028 duplicates, 50 studies were selected for full‐text review, and 15 met the eligibility criteria and were therefore included in the review. These studies, published between 2007 and 2024, were conducted in various countries, including Turkey (n = 3), China (n = 2), and one study each from Thailand, Hungary, Mexico, Colombia, Morocco, Pakistan, India, Romania, Brazil, and Argentina. Nine studies adopted a societal perspective, whereas six used a health care perspective. The total sample size was 218,575 participants, with individual study sizes ranged from 62 to 209,292. Average annual direct costs per patient ranged from US$523 to US$2,837.90, and indirect costs ranged from US$81.80 to US$2,463.40. The pooled average annual costs for outpatients, inpatients, and medical costs were US$517.72 (95% confidence interval [CI] $3.35–$1,032.09), US$543.88 (95% CI US$499.51–US$588.24), and US$3,379.83 (95% CI US$3,137.58–US$3,622.08), respectively.

**Conclusion:**

RA poses a significant economic challenge in LMICs, where limited health care resources and high treatment costs make care unaffordable for many. This review uniquely underscores that enhancing treatment access and optimizing resource use can reduce both medical and productivity losses, improving patient outcomes and strengthening economic resilience.

## INTRODUCTION

Rheumatoid arthritis (RA) is a chronic autoimmune disease characterized by persistent inflammation primarily affecting the joints but also impacting multiple extra‐articular organs such as the heart, kidneys, lungs, digestive system, and nervous system.^1^ RA leads to debilitating symptoms, including pain, stiffness, fatigue, and reduced mobility, ultimately causing long‐term disability and reduced quality of life.^2^ RA is among the most prevalent chronic diseases worldwide, impacting around 1% of the global population. It typically affects children and young adults aged 16 to 40 years, with a higher prevalence observed in industrialized countries.^3^ RA presents a significant societal burden due to its high rates of morbidity, disability, and economic costs.^4^

**SIGNIFICANCE & INNOVATIONS**

- This study addresses the underexplored economic burden of rheumatoid arthritis (RA) in low‐ and middle‐income countries (LMICs), offering a comprehensive understanding of its impact on households, health systems, and societies in resource‐limited settings, in contrast to the focus of much existing research on high‐income countries.
- The review adhered to Preferred Reporting Items for Systematic Reviews and Meta‐Analyses (PRISMA) guidelines and conducted a rigorous quality appraisal using the Newcastle–Ottawa Scale, incorporating studies from various LMICs to provide a comprehensive perspective on RA's economic burden and highlight regional differences in costs and access to care.
- The review offers data to guide policy decisions aimed at improving access to affordable treatments, optimizing health care resources, and boosting health outcomes for individuals with RA in LMICs.

The economic impact of RA can be categorized into direct, indirect, and intangible costs.^5^ Direct costs include expenses related to medical treatments, hospitalizations, medications, and transportation for health care services.^6^ Indirect costs encompass productivity losses from absenteeism or early retirement due to illness.^7^ Psychosocial or intangible costs, though challenging to quantify, represent the deterioration in the quality of life for patients, families, and caregivers.^8^ These components collectively form the economic burden of RA, which has been extensively studied in high‐income countries (HICs) but remains poorly understood in low‐ and middle‐income countries (LMICs).

In LMICs, the economic burden of RA is significant due to high treatment costs and loss of productivity. Limited health care resources and access to specialized care further exacerbate the financial strain on individuals and health care systems.^9^ Moreover, RA contributes to a larger global health challenge given that noncommunicable diseases (NCDs) increasingly affect the working‐age population in these countries.^10^ The rising prevalence of NCDs in LMICs, alongside the continued challenge of communicable diseases, results in a double burden that hampers national development and exacerbates poverty. For patients with RA, the inability to work due to disability can have devastating economic consequences, particularly in resource‐constrained settings in which there is minimal or no social support for individuals with disability.

Given the expanding burden of RA in LMICs, it is crucial to understand the economic impact of the disease in these regions. Cost‐of‐illness (COI) studies provide a comprehensive analysis of the financial burden by assessing the various cost components associated with the disease. These studies offer valuable insights into where the major costs lie, which can guide policy decisions, health care planning, and the allocation of resources for RA care and treatment. However, there is a paucity of data on the economic burden of RA in LMICs, which makes it challenging to fully appreciate the extent of the problem. This paper presents a systematic review of published COI studies on RA, focusing on the economic burden in LMICs. By highlighting the economic burden of RA, this review aims to support evidence‐based policymaking that can reduce the impact of the condition and improve health care delivery in LMICs, ultimately benefiting both patients with RA and the society.

## METHODS

A comprehensive systematic review was conducted in accordance with the Preferred Reporting Items for Systematic Reviews and Meta‐Analyses (PRISMA) guidelines, as outlined by Moher et al.^11^ This approach ensured a rigorous and transparent methodology for identifying, evaluating, and synthesizing relevant studies. Additionally, the review was formally registered with PROSPERO (PROSPERO is an international online registry where researchers prospectively record protocols for systematic reviews to promote transparency and reduce duplication), the International Prospective Register of Systematic Reviews, under Centre for Reviews and Dissemination registration number 42024566816, to ensure proper oversight and prevent duplication of efforts.

### Search strategy

The literature search for this systematic review was conducted using the PubMed, Web of Science, and CINAHL databases, with studies published from inception up until March 28, 2025, being included. The search terms used encompassed various combinations such as “burden of disease,” “length of stay,” “cost of illness,” “burden of illness,” “cost of disease,” “cost of sickness,” “disease cost,” “economic burden of disease,” “sickness cost,” “rheumatoid arthritis,” and “arthritis.” In addition to these databases, hand searches were conducted by reviewing the references of the included studies. The search terms were cross‐referenced with the Medical Subject Headings terms to ensure comprehensive coverage. All references were imported into Covidence. The search process was independently conducted by two reviewers (TG and FN) to minimize bias in the study selection and exclusion. Any disagreements were resolved through discussion with a third reviewer (CM).

### Inclusion and exclusion criteria

The reviewers independently selected publications for inclusion in this review based on predefined eligibility criteria. Any discrepancies in selection were resolved through consensus among the reviewers. The inclusion criteria specified that eligible studies must involve retrospective or prospective study designs conducted in primary or secondary care settings. Additionally, only studies reporting costs related to RA in LMICs, with study populations comprising patients with RA, and published in English were considered. Exclusion criteria encompassed studies unrelated to the economic consequences of RA, as well as personal papers, conference abstracts, case reports, letters, commentaries, editorials, review articles, and studies lacking sufficient data.

### Data extraction and quality assessment

**Figure 1:**
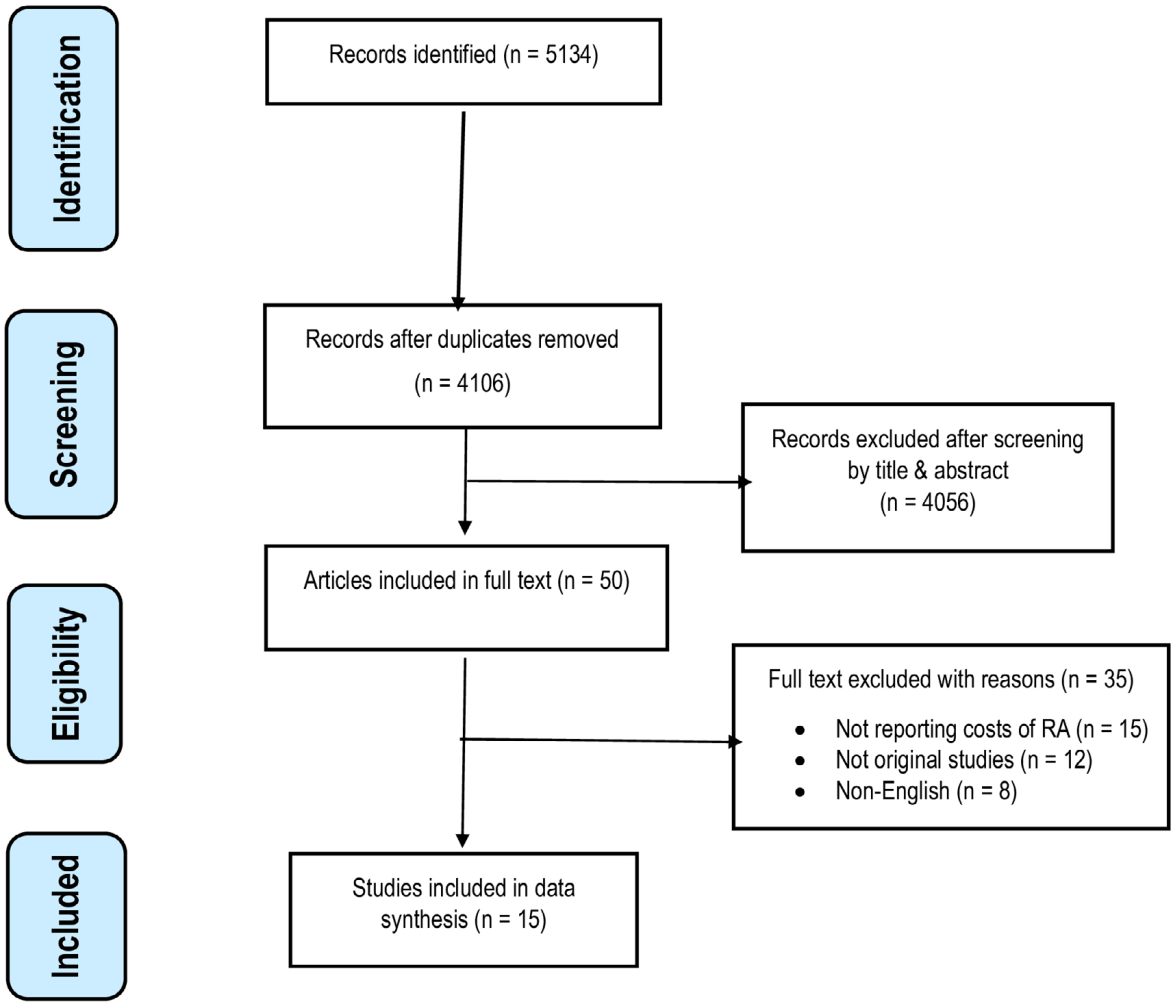
Flow diagram of publications included and excluded in the review. RA, rheumatoid arthritis.

The study selection process is detailed in the PRISMA flow diagram (Figure 1). Data extraction was conducted independently by two reviewers (TG and FN) using a standardized Excel‐based data extraction form. To ensure accuracy, a third reviewer (CM) verified the extracted data. Information extracted from the included studies encompassed author and year of publication, study setting, cost perspective, data sources, outcome measures, sample size, year of costing, and various cost categories such as inpatient, outpatient, drug/medical, nonmedical, direct, indirect, and total costs. Supplementary Table 1 presents the definitions and classifications of health care–related costs.^12^ For cost‐related data, the currency, cost year, and the reported mean or median total and RA‐attributable costs were also recorded. Additionally, we extracted information on the epidemiologic approach used in each study. In COI analysis, this includes two main perspectives: the incidence‐based approach, which estimates the lifetime costs of newly diagnosed cases to inform potential preventive savings, and the prevalence‐based approach, which assesses the current total costs associated with all existing cases over a defined period.^13^

The quality of the included studies was assessed using the Newcastle–Ottawa Scale (NOS) by two reviewers (TG and FN). The NOS evaluates studies across nine criteria, categorized into three dimensions: selection of the study population, comparability of groups, and assessment of outcomes or exposures.^14^ Studies were scored on a scale with a maximum of nine points, in which a score of ≥6 indicated high quality, a score between 3 and 6 indicated moderate quality, and a score of ≤3 indicated low quality. Any disagreements in scoring were resolved through consultation with a third reviewer (CM).

### Data analysis

In this study, summary descriptive statistics were employed to characterize the study background and the types of costs associated with RA. Both direct and indirect costs related to RA were further examined through quantitative analysis. A cross‐country cost comparison was conducted based on the methodologies used in the included studies. To facilitate this comparison, costs were converted to US dollars (US$) using country‐specific gross domestic product (GDP) deflators^15^ and purchasing power parities (PPPs).^16^ These conversions were performed as of December 2024. Estimated cost values were adjusted by multiplying them by the 2024 GDP coefficient, then dividing by the GDP of the reference year for each study and finally adjusted by the PPP conversion factor for 2024.

A meta‐analysis was conducted to synthesize the cost estimates for RA from multiple studies. The analysis included studies reporting mean costs, SDs, and sample sizes. For each study, the SE was calculated using the formula SE = SD/√n, and 95% confidence intervals (CIs) were derived as mean ± 1.96 × SE. An inverse‐variance weighted approach was applied to compute a pooled mean estimate, giving greater weight to studies with larger sample sizes and lower variance. Forest plots were generated to visually present individual study estimates alongside the pooled estimate, including corresponding 95% CIs.

## RESULTS

A total of 5,134 studies were initially identified for screening, including 3,599 from Web of Science, 788 from CINAHL, and 747 from PubMed. After removing 1,028 duplicates, the remaining studies were screened by title and abstract, resulting in 50 studies selected for full‐text review. Of these, 15 studies ultimately met the predefined eligibility criteria for inclusion. The majority of studies included in this systematic review were of moderate to high quality based on the NOS score (Supplementary Table 2).

### Characteristics of the included studies

The characteristics of the included studies are presented in Table 1. The included studies estimated the COI of RA in 12 countries. These studies were published between 2007 and 2024. The total sample size across these studies was 218,575 participants; individual study sample sizes ranged from 62 to 209,292. The majority were conducted in Asia^17^, ^18^, ^19^, ^20^, ^21^ followed by Europe,^22^, ^23^, ^24^, ^25^, ^26^ Latin America,^27^, ^28^, ^29^, ^30^ and Africa.^31^ Among the studies, female participants accounted for the main composition of the population, ranging from 42% to 92%. Of the 15 studies, 9 adopted a societal perspective, whereas 6 used a health care perspective.

**Table 1:** Characteristics of the included studies*

Author	Setting, year of costing	Method and sample description	Cost perspective	Source of data	Type of outcomes calculated	NOS score
Baser et al^23^	Turkey, 2013	Incidence and prevalence based, 2,613 (693 were incident cases and 1,920 prevalent cases)	Health care perspective	The Turkish National Health Insurance Database (2009–2011)	Annual costs for incident and patients with prevalent RA after controlling for age, gender, region, comorbid conditions, and medication Total health care costs	6
Ayan et al^24^	Turkey, 2018	Prevalence based, 62 (82.2 women)	Societal perspective	Multicenter study assessing RA‐related health care costs in Turkey that was performed between May 2011 and August 2012	Direct and indirect costs	6
Hamuryudan et al^26^	Turkey, 2011	Prevalence based, 689	Societal perspective	The rheumatology outpatient clinics of 10 tertiary care hospitals in Turkey	Direct and indirect costs of RA	7
Horváth et al^22^	Hungary, 2012	Incidence based, 209,292 patients (225,623 cases)	Payer's perspective	National Health Insurance Fund Administration	Number of patients and cases, crude and weighted hospital days, and health insurance expenditure	7
Codreanu et al^25^	Romania, 2014	Prevalence‐based/self‐reported questionnaires, 206, mean age 54.9 ± 12.7 years	Societal perspective	The electronic database of a university tertiary rheumatology center	Indirect costs	7
Fellous et al^31^	Morocco, 2020	Incidence based, 197 (86.8% women)	Health care perspective	The Moroccan registry of biologic therapies in RA	Direct medical costs	7
Osiri et al^17^	Thailand, 2007	Prevalence based, 158 (>90% women)	Societal perspective	From 158 patients with RA who attended a major tertiary care facility in Bangkok, Thailand	Direct medical, direct nonmedical, indirect, and total costs	6
Hu et al^18^	China, 2013	Prevalence based, 133 (70.68% women)	Societal perspective	The COI questionnaire	Direct, indirect, and intangible costs	6
Xu et al^21^	China, 2009	Prevalence based, 829 (78.6% women)	Societal perspective	Data collected by trained physicians using a face‐to‐face interview with patients	Direct and indirect costs of RA	6
Naqvi et al^19^	Pakistan, 2019	Prevalence based, 358 (73.7% women)	Health care perspective	Rheumatology departments of two public sectors and three private sector tertiary care hospitals	Direct costs	5
Bali and Singla,^20^	India, 2022	Prevalence based/cross‐sectional, 67 (89.5% women)	Societal perspective	Data collected over a two‐month period from June to July 2022 from patients attending the weekly rheumatology OPD	Direct and indirect costs	5
Mendoza‐Gutierrez et al^30^	Mexico, 2016 and 2017	Prevalence based, in 2016 and 2017, a total of 3,623 (2,944 women [81.3%] and 3,427 2,779 women [81.1%], respectively)	Payer's perspective	Mexican Social Security Institute	Direct economic costs	5
Santos‐Moreno et al^27^	Colombia, 2019	Prevalence based, 83 patients (42% women)	Health care perspective	A rheumatology care center in Bogotá	Direct medical costs	6
Chermont et al^28^	Brazil, 2002	Prevalence based, 100 (92% women)	Societal perspective	The outpatient clinics for RA at the Division of Rheumatology of the Federal University of São Paulo	Direct and indirect costs	6
Catay et al^29^	Argentina, 2002	Prevalence based, 165 (84 women)	Societal perspective	Health management organization administrative and medical databases and questionnaire	Direct and indirect costs	7

* Incidence‐based COI estimates the lifetime costs associated with new cases of a disease within a specific time period. Prevalence‐based COI estimates the total costs of all existing cases of a disease within a defined population and time period. COI, cost of illness; NOS, Newcastle–Ottawa Scale; OPD, outpatient department; RA, rheumatoid arthritis.

In eight of the included studies^20^, ^22^, ^23^, ^24^, ^26^, ^27^, ^29^, ^31^ the data sources were retrospective databases including health insurance databases, disease registries, and hospital administrative records. The remaining studies described results from self‐reported questionnaire surveys.^17^, ^18^, ^19^, ^21^, ^25^, ^28^, ^30^ Most included studies^17^, ^18^, ^19^, ^20^, ^21^, ^24^, ^25^, ^26^, ^27^, ^28^, ^29^, ^30^ were conducted using a prevalence‐based approach. The studies that adopted the incidence‐based approach^22^, ^23^, ^31^ focused on patients with recent onset. Cost components and measurement of direct or indirect costs also varied markedly due to the aims and data availability among studies. Nine of the included studies,^17^, ^18^, ^20^, ^21^, ^24^, ^25^, ^26^, ^27^ reported both direct and indirect costs, whereas the remaining studies reported only direct costs.

### Medical and nonmedical costs of RA

Table 2 presents the medical and nonmedical costs of RA. In estimating the direct costs associated with RA, various cost components were incorporated into the analysis. These typically included drug‐related expenses, inpatient care costs, outpatient care costs, and other health care–associated expenditures. Nine of the studies included in the analysis provided data on the annual medical costs, which ranged from US$1,728 to US$7,584 per patient per year.^16^, ^17^, ^18^, ^19^, ^21^, ^24^, ^26^, ^27^, ^28^, ^29^ Additionally, six studies reported the nonmedical costs of RA, which ranged from US$319 to US$7,988 per patient per year.^15^, ^17^, ^20^, ^21^, ^22^, ^28^, ^29^ These figures highlight the substantial financial burden of RA, encompassing both direct medical costs and other related expenditures.

**Table 2:** Medical and nonmedical costs of RA*

Author, country, year of costing	Inpatient costs, mean (±SD), inflated 2024 US$	Outpatient costs, mean (±SD), inflated 2024 US$	Medical costs, mean (±SD), inflated 2024 US$	Nonmedical costs, mean (±SD), inflated 2024 US$
Baser et al,^23^ Turkey, 2013	US$335 (±US$451) for prevalent cases and US$480 (±US$646) incident cases per year	US$478.64 (±US$645) for prevalent and $547.61 (±US$737) for incident cases per year	NR	NR
Ayan et al,^24^ Turkey, 2018	US$145,969 (±US$182.30), 95% CI US$122.04 (±US$152.30)–US$410,389 (±US$512.20) per patient per year	US$26,3223 (±US$32.90), 95% CI US$13.20 (±US$16.50)–US$40.68 (±US$50.80) per patient per year	US$3,539.16 (±US$4,418), 95% CI US$120.80 (±US$150.70)–US$4,361.13 (±US$5,444) per patient per year	NR
Hamuryudan et al,^26^ Turkey, 2011	Mean ± SD US$195 (US$272), US$857 (US$1,194) per patient per year	Mean ± SD US$382 (US$532), US$428 (US$597) per patient per year	Mean ± SD US$2,777 (US$3,872), US$3,379 (US$4,711) per patient per year	NR
Horváth et al,^22^ Hungary, 2012	NR	NR	NR	Day care: US$1.18 million (±US$1.60 million) Nursing: US$6.56 million (±US$8.86 million) Long‐term care: US$30.15 million (±US$40.79 million) Rehabilitation: US$108.81 million (±US$147.08 million)
Osiri et al,^17^ Thailand, 2007	NR	NR	Mean US$1,923 (±US$2,878), median US$1,036 (US$1,550) per patient per year	Mean US$213 (±US$ 319), median US$89 (US$ 133) per patient per year
Hu et al,^18^ China, 2013	Mean US$593.03 (±US$798), US$1,220.35 (±US$1,641) per patient per year	Mean US$1,324.18 (±US$1,781), US$1,990.17 (±US$2,677) per patient per year	Mean US$1,283.89 (±US$1,728), US$1,898.15 (±US$2,553) per patient per year	NR
Xu et al,^21^ China, 2009	NR	NR	Mean US$3,211 (±US$4,695), US$5,264 (±US$7,692) per patient per year	Mean US$232 (±US$339), US$662 (±US$967) per patient per year
Naqvi et al,^19^ Pakistan, 2019	NR	NR	Medicines: US$63.25 (±US$73.79) per patient per year Medical devices costs: US$49.13 (±US$57.23) per patient per year Rheumatologist visits: US$72.05 (±US$83.97) per patient per year Physical therapy sessions: US$419.07 (±US$488.87) for per patient per year	NR
Bali and Singla,^20^ India, 2022	NR	NR	NR	Out‐of‐pocket expenses, mean (±SD): US$230 (±US$259.67)
Mendoza‐Gutierrez et al,^30^ Mexico, 2016 and 2017	US$9,096,245.67 (±US$12,280,932.64) for 2016 and US$8,932,592.57 (±US$11,612,370.34) for 2017 (total cost per year)	NR	NR	NR
Santos‐Moreno et al,^27^ Colombia, 2019	NR	NR	Total = Mean (±SD) US$6,067 (±US$7,584), US$6,144 (±US$7,680) per patients per year Male: Mean (± SD) US$6,160 (±US$7,700), US$5,959 (±US$7,449) Female: Mean (±SD) US$5,893 (±US$7,366), US$6,545 (±US$8,181)	NR
Chermont et al,^28^ Brazil, 2002	NR	NR	US$370.36 (±US$1,259.22) per patient per year	US$32.68 (±US$111.11) per patient per year
Catay et al,^29^ Argentina, 2002	NR	NR	US$1,862 (±US$6,735)…US$67,032 (±US$242,460) per patient per year	US$221.90 (±US$315)…US$7,988 (±SD US$11,340) per patient per year

* CI, confidence interval; NR, not reported; RA, rheumatoid arthritis; US$, US dollars.

### Meta‐analysis

**Figure 2:**
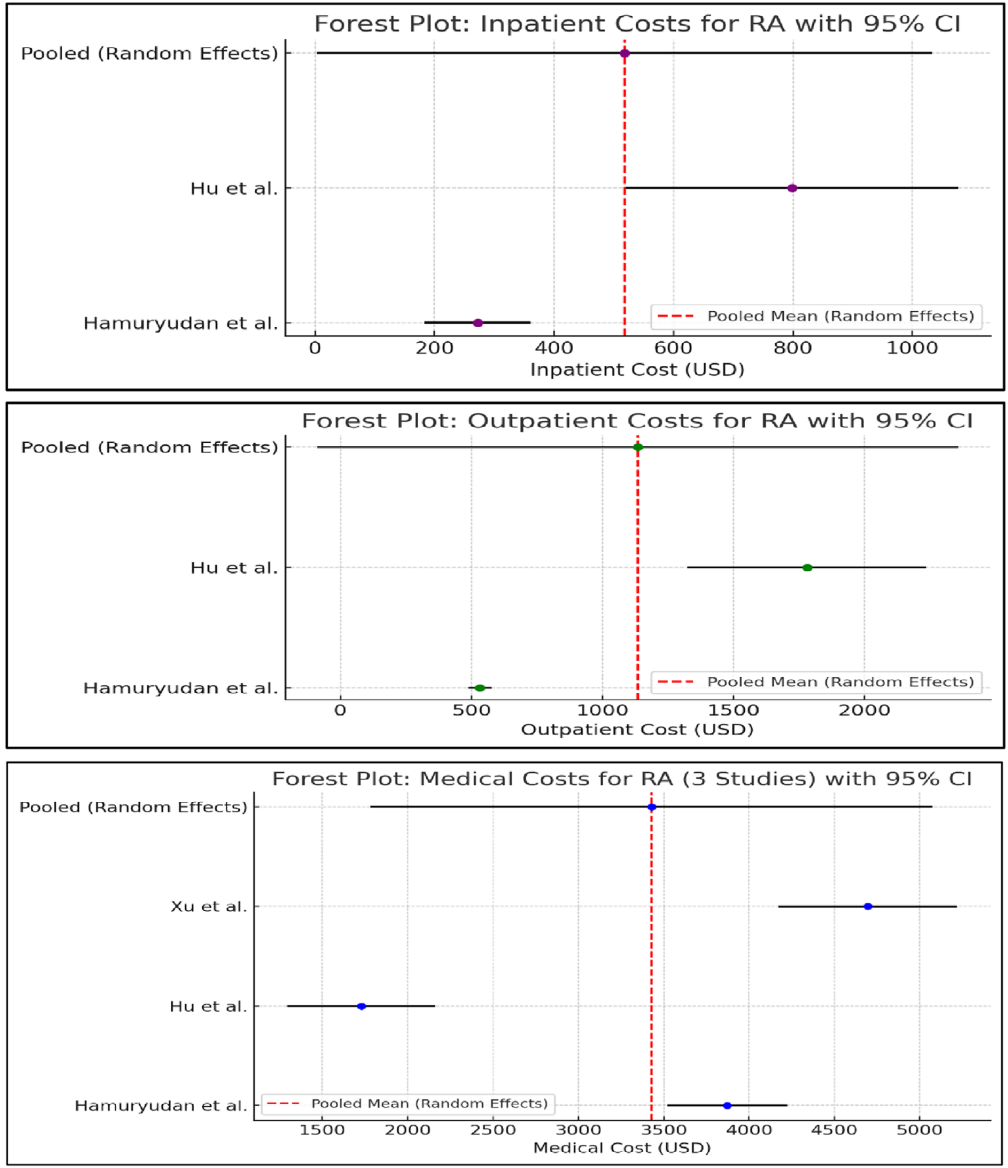
Forest plot of inpatient, outpatient, and medical costs for RA with 95% CI. CI, confidence interval; RA, rheumatoid arthritis. Color figure can be viewed in the online issue, which is available at http://onlinelibrary.wiley.com/doi/10.1002/acr.25627/abstract.

The meta‐analysis of inpatient, outpatient, and medical costs for RA with 95% CI, including the pooled random‐effects estimate, is presented (Figure 2). The pooled average annual costs for outpatients, inpatient, and medical costs were US$517.72 (95% CI US$3.35–US$1,032.09), US$543.88 (95% CI US$499.51–US$588.24), and US$3,379.83 (95% CI US$3,137.58–US$3,622.08), respectively.

### Direct and indirect costs of RA

The direct and indirect costs associated with RA are summarized in Table 3. Ten of the studies included in the analysis reported the average annual direct costs of RA, which varied widely, ranging from US$523 to US$2,837.90.^17^, ^18^, ^19^, ^20^, ^21^, ^22^, ^23^, ^24^, ^28^, ^31^ In terms of indirect costs, seven studies provided estimates, with values spanning from US$90.59 to US$3,697 per patient per year.^25^, ^26^ Notably, one study^21^ that assessed the annual direct and indirect costs of RA in China found that direct costs accounted for 90% of the total costs, whereas indirect costs made up the remaining 10%. This highlights the significant contribution of direct medical expenses to the overall economic burden of RA, though indirect costs, such as productivity loss, remain a notable cost driver.

**Table 3:** Direct and indirect costs of RA*

Author, country, year of costing	Direct costs	Direct costs, inflated 2024 US$	Indirect costs	Indirect costs, inflated 2024 US$	Total costs	Total costs, inflated 2024 US$
Baser et al,^23^ Turkey, 2013	US$2,660 per patient, 95% CI US$2,327.50–US$2,990.51	US$3,485 per patient, 95% CI US$3,049–US$3,918	NR	NR	NR	NR
Ayan et al,^24^ Turkey, 2018	Median (range) US$3,538 (US$124–US$4,353) per patient per year	Median (range) US$20,167 (US$707–US$24,812) per patient per year	Median (range) US$359 (US$124–US$1,186) per patient per year	NR	NR	NR
Horváth et al,^22^ Hungary, 2012	US$148 million per year	US$198.30 million	NR	NR	NR	NR
Codreanu et al,^25^ Romania, 2014	NR	NR	US$2,720 per patient per year	US$3,697 per patient per year	NR	NR
Fellous et al,^31^ Morocco, 2020	Total annual cost = US$1,115,483 (drug prices, cost of infusions, cost of subcutaneous injection) Median (range) US$1,898 (US$1,678–US$11,272) per patient per year	Total annual cost ≈ US$1.25 million Median (range) US$2,132 (US$1,885–US$12,642) per patient per year	NR	NR	NR	NR
Osiri et al,^17^ Thailand, 2007	Mean US$2,135 (median US$1,186) per patient per year	Mean US$2,837.90, (median US$1,576.50) per patient per year	Mean US$547 (median US$182) per patient per year	Mean US$3,564 (median US$1,183) per patient per year	Mean US$2,682 (median US$1,434) per year	Mean US$3,565 (median US$1,906) per patient per year
Hu et al,^18^ China, 2013	Mean (±SD) US$1,917.21 (±US$2,559.06) per patient per year	Mean (±SD) US$2,320 (±US$3,098) per patient per year	Mean (±SD) US$492.88 (±US$1,739.74) per patient per year	Mean (±SD) US$596.39 (±US$2,106.08) per patient per year	NR	NR
Xu et al,^21^ China, 2009	90.0% of the total costs	90.0% of the total costs	10.0% of the total costs	10.0% of the total costs	Mean (±SD) US$3,826 (±US$5,659) per patient per year	Mean (±SD) US$5,470.18 (±US$8,095.37) per patient per year
Naqvi et al,^19^ Pakistan, 2019	Mean US$235.10 per patient per year	Mean US$523 per patient per year	NR	NR	NR	NR
Bali and Singla,^20^ India, 2022	Mean (±SD) US$424 (±US$634) per patient per year	Mean (±SD) US$572 (±US$856) per patient per year	Mean (±SD) US$226 (US$273) per patient per year	Mean (±SD) US$252 (±US$302) per patient per year	Mean (±SD) US$652 (±US$746) per patient per year	Mean (±SD) US$724 (±US$828) per patient per year
Mendoza‐Gutierrez et al,^30^ Mexico, 2016 and 2017	NR	NR	Mean (±SD) US$1,677 (±US$4,180); Caregiver US$640 (±US$747); Workday loss US$485 (±US$1,386) per patient per year	NR	NR	NR
Chermont et al,^28^ Brazil, 2002	Mean US$382.89 per patient per year	Mean US$1,723 per patient per year	Mean US$20.13 per patient per year	Mean US$90.59 per patient per year	Mean US$403.04 per patient per year	Mean US$1,813.68 per patient per year
Catay et al,^29^ Argentina, 2002	NR	NR	Mean (±SD) US$1,008.80 (±US$1,913) per patient per year	Mean (±SD) US$1,712.12 (±US$3,248.54) per patient per year	US$3,093, 95% CI 1,581–4,605 per patient per year	Mean US$5,250.72, 95% CI US$2,683.56–US$7,818.06 per patient per year

* CI, confidence interval; NR, not reported; RA, rheumatoid arthritis; US$, US dollars.

## DISCUSSION

This systematic review offers an original contribution by assessing the economic burden of RA in LMICs, a topic with limited existing synthesis. Drawing on 15 studies from 12 countries, the review reveals a substantial economic impact, with wide variation in direct and indirect costs influenced by study design, health care system differences, and data collection methods. The findings highlight critical patterns and gaps that are essential for policymakers, health care providers, and researchers working to address RA in LMICs.

As stated above, this review highlights significant variation in both direct and indirect costs associated with RA. Direct costs ranged from US$523 to US$2,837.90 per patient annually, whereas indirect costs varied from US$81.80 to US$2,463.40. These discrepancies are likely attributed to differences in health care systems, economic conditions, and research methodologies across countries, especially in LMICs, where patients often face long travel distances, high out‐of‐pocket expenses, and limited access to medications and diagnostic tools compared to high‐income nations.^32^ The structure of health care systems also influences these costs, with advanced health care systems typically incurring higher direct costs. Indirect costs, representing productivity losses from patients and caregivers, are harder to quantify and tend to be lower due to broader societal factors.^33^ Methodologic differences in studies often make direct costs more prominent because they are easier to measure than the more elusive indirect costs.^34^

The review also found substantial variation in the study designs and methods used to estimate costs. Nine studies adopted a societal perspective, whereas six used a health care perspective. The societal perspective, which includes both direct and indirect costs, provides a more holistic view of the economic burden of RA.^35^ However, the health care perspective is often more feasible to implement, especially in LMICs with limited resources for large‐scale societal surveys.^36^ The most commonly used data sources were retrospective databases such as health insurance databases and disease registries, followed by self‐reported surveys. The reliance on retrospective data and self‐reported questionnaires introduces the potential for bias and inaccuracies in the cost estimates, which must be taken into account when interpreting the results.

This review shows that RA places a heavy economic burden on individuals, families, health systems, and society in LMICs, and governments need to take clear, practical steps to address it. The significance of our findings is that RA should be included in national plans for NCDs to ensure adequate resources for prevention, early diagnosis, and treatment.^37^ In addition, countries also need national RA registries to track disease prevalence, treatment outcomes, and the costs involved.^38^ Moreover, training more health care workers to diagnose RA early can help patients get treatment sooner, reducing disability and health care costs in the long run.^39^ Expanding health insurance coverage to include RA services, as well as offering rehabilitation and programs that help patients stay in the workforce, can ease the economic burden on both individuals and society.^3^

We also need the support of HICs and global organizations to reduce the impact of RA in LMICs. HICs can help by providing funding, technical expertise, and resources to build reliable health data systems and national RA registries.^40^ Additionally, international partnerships should focus on training health care workers and policymakers in early diagnosis, affordable treatment options, and how to measure health care costs effectively.^39^ It is also important for global research projects to include LMICs so that studies produce evidence relevant to these settings.^41^ Finally, international agencies and HIC governments should push to ensure RA and other musculoskeletal conditions are part of global NCD programs, so they get the attention and resources they deserve.^42^

This systematic review has both strengths and limitations. Its strength lies in the comprehensive approach taken to synthesize existing evidence, offering a deep understanding of the economic burden of RA. The review's originality is highlighted by its focus on LMICs, where the economic impact of RA is often underexplored, providing valuable insights for policy development in these regions. We also adhered to a standardized and widely accepted reporting framework for systematic reviews and meta‐analyses to ensure transparency, consistency, and methodologic rigor throughout the review process. However, one limitation is the substantial heterogeneity across the included studies, particularly regarding data sources and methodologies, which makes it challenging to draw definitive conclusions about the overall economic burden of RA in LMICs. Only studies published in English language were included. Therefore, it is possible that relevant studies published in other languages may have been excluded. Lastly, although the studies included were of moderate to high quality, the reliance on retrospective data and self‐reported questionnaire may introduce biases, potentially affecting the accuracy of the cost estimates.

In conclusion, this systematic review highlights the significant economic burden of RA on households, health systems, and society in LMICs, with both direct and indirect costs imposing considerable strain on individuals, families, and health care systems. The substantial variability in cost estimates underscores the need for more standardized, high‐quality studies to better understand the full scope of RA's economic impact. Given the rising prevalence of RA and its associated costs, the significance of our findings is that there is a pressing need for targeted policies and interventions to reduce the burden of this disease in low‐resource settings.

## AUTHOR CONTRIBUTIONS

All authors contributed to at least one of the following manuscript preparation roles: conceptualization AND/OR methodology, software, investigation, formal analysis, data curation, visualization, and validation AND drafting or reviewing/editing the final draft. As corresponding author, Dr Gebrye confirms that all authors have provided the final approval of the version to be published and takes responsibility for the affirmations regarding article submission (eg, not under consideration by another journal), the integrity of the data presented, and the statements regarding compliance with institutional review board/Declaration of Helsinki requirements.

## Supporting information


**Disclosure Form**:

**Supplementary Table 1:** Definitions and classifications of healthcare‐related costs
**Supplementary Table 2:** Risk of bias assessment of the included studies
