# Supplementary material for: Economic Burden of Rheumatoid Arthritis in Low‐ and Middle‐Income Countries: Systematic Review and Meta‐Analysis
Source: Arthritis Care Res (Hoboken). 2025 Nov 22;78(3):325–36. doi: 10.1002/acr.25627 (PMC12975684; doi:10.1002/acr.25627)
Supplement: Supplementary file 2 — Supplementary Table 1: Definitions and classifications of healthcare‐related costs Supplementary Table 2: Risk of bias assessment of the included studies [file ACR-78-325-s001.docx]

**Supplementary Table 1:** Definitions and classifications of healthcare-related costs

| Category | Definition | Classifications |
| --- | --- | --- |
| Outpatient Costs | Expenses related to medical care or procedures that do not require hospital admission. | Doctor visits, diagnostic tests, minor surgical procedures, physical therapy, outpatient drugs |
| Medical Costs | All costs directly related to the prevention, diagnosis, treatment, and rehabilitation of health conditions. | Hospital stays, physician services, surgeries, medications, diagnostics, medical devices |
| Non-Medical Costs | Costs incurred due to healthcare needs but not directly related to the provision of medical services. | Transportation, lodging, meals during treatment, caregiver time (unpaid), childcare |
| Direct Costs | Costs that are directly associated with the treatment and care of a patient, both medical and non-medical. | Medical: surgery, medication; non-medical: travel expenses, home modifications |
| Indirect Costs | Economic consequences of illness that affect productivity or income, often due to time lost from work or premature death. | Lost wages, decreased productivity, long-term disability, early retirement |

**Supplementary Table 2:** Risk of bias assessment of the included studies

| Studies | Selection 1* | Selection 2* | Selection 3* | Selection 4* | Comparability 1** | Exposure 1* | Exposure 2* | Exposure 3* | Total (MAX. 9) |
| --- | --- | --- | --- | --- | --- | --- | --- | --- | --- |
| Horváth et al. ^22^ | 1 | 1 | 1 | 1 | 0 | 1 | 1 | 0 | 6 |
| Baser et al.^23^ | 1 | 1 | 1 | 0 | 2 | 1 | 1 | 0 | 7 |
| Osiri et al. ^17^ | 1 | 1 | 1 | 0 | 0 | 1 | 1 | 0 | 5 |
| Hu et al.^18^ | 1 | 1 | 1 | 0 | 0 | 1 | 1 | 0 | 5 |
| Mendoza-Gutierrez et al.^30^ | 1 | 1 | 1 | 0 | 2 | 1 | 1 | 0 | 7 |
| Xu et al. ^21^ | 1 | 1 | 1 | 0 | 0 | 1 | 1 | 0 | 5 |
| Santos-Moreno et al. ^27^ | 1 | 1 | 1 | 1 | 0 | 1 | 1 | 0 | 6 |
| Fellous et al.^31^ | 1 | 1 | 1 | 0 | 0 | 1 | 1 | 0 | 5 |
| Hamuryudan et al. ^26^ | 1 | 1 | 1 | 0 | 0 | 1 | 1 | 0 | 5 |
| Naqvi et al. ^19^ | 1 | 1 | 1 | 0 | 0 | 1 | 1 | 0 | 5 |
| Ayan et al. ^24^ | 1 | 1 | 1 | 0 | 2 | 1 | 1 | 0 | 7 |
| Bali & Singla, ^20^ | 1 | 1 | 1 | 0 | 0 | 1 | 1 | 0 | 5 |
| Codreanu et al.^25^ | 1 | 1 | 1 | 0 | 0 | 1 | 1 | 0 | 5 |
| Chermont et al. ^28^ | 1 | 1 | 1 | 0 | 0 | 1 | 1 | 0 | 5 |
| Catay et al.^29^ | 1 | 1 | 1 | 0 | 2 | 1 | 1 | 0 | 7 |
